# PTGES is involved in myofibroblast differentiation via HIF‐1α‐dependent glycolysis pathway

**DOI:** 10.1111/jcmm.70157

**Published:** 2024-10-17

**Authors:** Min‐Hsi Lin, Yi‐Chen Lee, Jia‐Bin Liao, Chih‐Yu Chou, Yi‐Fang Yang

**Affiliations:** ^1^ Division of Chest Medicine Kaohsiung Veterans General Hospital Kaohsiung Taiwan; ^2^ Department of Anatomy, School of Medicine, College of Medicine Kaohsiung Medical University Kaohsiung Taiwan; ^3^ Department of Pathology and Laboratory Medicine Kaohsiung Veterans General Hospital Kaohsiung Taiwan; ^4^ Department of Medical Education and Research Kaohsiung Veterans General Hospital Kaohsiung Taiwan

**Keywords:** fibroblast, HIF‐1α, metabolic pathway, myofibroblast, PTGES

## Abstract

Lung cancer is the leading cause of cancer‐related deaths worldwide. Patients with lung cancer usually exhibit poor prognoses and low 5‐year survival rates. Idiopathic pulmonary fibrosis (IPF) and chronic obstructive pulmonary disease (COPD) are both chronic lung dysfunctions resulting in lung fibrosis and increased risk of lung cancer. Myofibroblasts contribute to the progression of asthma, COPD and IPF, leading to fibrosis in the airway and lungs. A growing body of evidence demonstrates that metabolic reprogramming is a major hallmark of fibrosis, being important in the progression of fibrosis. Using gene expression microarray, we identified and validated that the lipid metabolic pathway was upregulated in lung fibroblasts upon interleukin (IL)‐4, IL‐13 and tumour necrosis factor (TNF)‐α treatment. In this study, we described that prostaglandin E synthase (*PTGES*) was upregulated in lung fibroblasts after IL‐4, IL‐13 and TNF‐α treatments. PTGES increased α‐SMA levels and promoted lung fibroblast cell migration and invasion abilities. Furthermore, PTGES was upregulated in a lung fibrosis rat model in vivo. PTGES increased AKT phosphorylation, leading to activation of the HIF‐1α‐glycolysis pathway in lung fibroblast cells. HIF‐1α inhibitor or 2‐DG treatments reduced α‐SMA expression in recombinant PTGES (rPTGES)‐treated lung fibroblast cells. Targeting PGE_2_ signalling in PTGES‐overexpressing cells by a PTGES inhibitor reduced α‐SMA expression. In conclusion, the results of this study demonstrate that PTGES increases the expression of myofibroblast marker via HIF‐1α‐dependent glycolysis and contributes to myofibroblast differentiation.

## 1 INTRODUCTION

Lung cancer is among the most common malignancies not only in Taiwan but also in various other countries, and it remains the leading cause of cancer‐related deaths with an annual mortality rate beyond 1.3 million.^1^ Moreover, lung diseases, including asthma, idiopathic pulmonary fibrosis (IPF) and chronic obstructive pulmonary disease (COPD), are related to lung cancer development.^2^, ^3^, ^4^ However, the mechanism by which lung diseases mediate lung cancer development remains to be elucidated.

Fibroblasts are non‐vascular, non‐epithelial and non‐inflammatory cells and stroma‐forming components in the connective tissue.^5^, ^6^ Fibroblast functions include extracellular matrix (ECM) deposition, epithelial differentiation and inflammation regulation and involvement in wound healing.^7^, ^8^ In the lungs, fibroblasts are important in maintaining the integrity of the alveolar structure by proliferating and repairing the injured areas.^9^ Pathological fibroblast accumulation is characterized by fibrotic lung diseases.^10^ Increased airway fibroblast numbers in the submucosa contribute to the progression of subepithelial fibrosis during airway remodelling in asthma.^11^ Furthermore, COPD and IPF display similar phenomena wherein fibroblast and myofibroblast accumulation along with excessive ECM production promotes fibrosis formation.^12^, ^13^ Lung diseases, including asthma, IPF and COPD, also participate in lung cancer progression.^2^, ^3^, ^4^ However, the underlying mechanism by which lung fibrosis is activated and recruited remains elusive.

When tissues are injured, the residential fibroblasts differentiate into myofibroblasts upon paracrine signals.^14^ Fibroblast‐to‐myofibroblast transition involves two stages. The first stage comprises fibroblast transition into proto‐myofibroblasts induced by mechanical tension within the wound, along with platelet‐derived growth factor (PDGF) secretion. PDGF induces fibre formation and increases cell motility.^15^ The second stage corresponds to proto‐myofibroblast transition into myofibroblasts triggered by cytokines, growth factors and ECM proteins, leading to α‐smooth muscle actin (α‐SMA) synthesis and gradual α‐SMA‐containing stress fibre formation.^16^ Previous studies have demonstrated that normal myofibroblasts can reversibly differentiate into fibroblasts in vitro.^17^, ^18^ Myofibroblasts could cause organ fibrosis, thereby increasing the risk of cancer.^14^, ^19^ Furthermore, myofibroblasts, also known as cancer‐associated fibroblasts (CAFs), are abundant in various tumour microenvironments (TME).^20^ CAFs could derive from endothelial, smooth muscle, myoepithelial or mesenchymal stem cells and secrete growth factors, which are mitogenic for malignant cells.^21^, ^22^, ^23^, ^24^ CAFs are activated fibroblasts that promote tumour initiation,^25^ progression,^26^ and metastasis^27^ by recruiting and communicating with cancer cells.^28^ An increasing body of evidence demonstrates that CAFs could be activated by factors in the TME, such as transforming growth factor‐β (TGF‐β), to become myofibroblasts (α‐SMA+/vimentin).^29^ CAFs promote tumour development via cell–cell interactions or cross‐talk with tumour cells by secreting growth factors, cytokines and exosomes.^30^ However, the mechanism by which CAFs are activated and recruited remains unclear.

Fibrosis could lead to organ dysfunction, morbidity and death upon abnormal ECM deposition. Metabolic alterations reportedly represent an important pathogenic process of fibrosis across various organ types.^31^ In fibrosis disorders, such as cirrhosis, renal fibrosis and IPF, glycolysis is upregulated, thereby contributing to fibroblast activation.^32^, ^33^, ^34^ Treatment with metformin, an antidiabetic drug that inhibits gluconeogenesis, induces lipogenic differentiation in myofibroblasts to reverse lung fibrosis.^35^ Previous studies have demonstrated that interleukin (IL)‐4, IL‐13 and tumour necrosis factor (TNF)‐α induce the myofibroblast phenotype of lung fibroblasts.^36^, ^37^ Myofibroblasts play are important in lung disease development, including asthma, COPD and IPF.^38^, ^39^ However, the interplay among cytokines and their effect on lung diseases remains unclear. Therefore, in our study, we analysed differentially expressed genes upon each cytokine treatment in lung fibroblast cells. We focused on the overlapping IL‐4‐, IL‐13‐ and TNF‐α‐induced gene expression patterns. Moreover, metabolic reprogramming is a reported major hallmark of fibrosis^31^ to the advantage of cancer cells.^39^ Hence, we observed PTGES, a lipid metabolic enzyme, to elucidate the interplay between PTGES and lung fibrosis development and progression.

PTGES (as PGE_2_ synthase enzyme) catalyzes endoperoxide PGH_2_ conversion to PGE_2_ and it is present and upregulated both in inflammatory tissues and cancer. In the tumour microenvironment, PGE_2_ plays a role in promoting immunosuppression.^40^, ^41^ In this study, we observed that PTGES promotes fibroblast transformation into myofibroblasts. PTGES induced AKT phosphorylation, resulting in increased HIF‐1α expression, thereby promoting glycolysis in myofibroblasts, which are critical stromal components in cancer cell progression.

## 2 MATERIALS AND METHODS

### 2.1 In silico *PTGES* , *ST8SIA1* and *PTGS2* mRNA profiles

**FIGURE 1:**
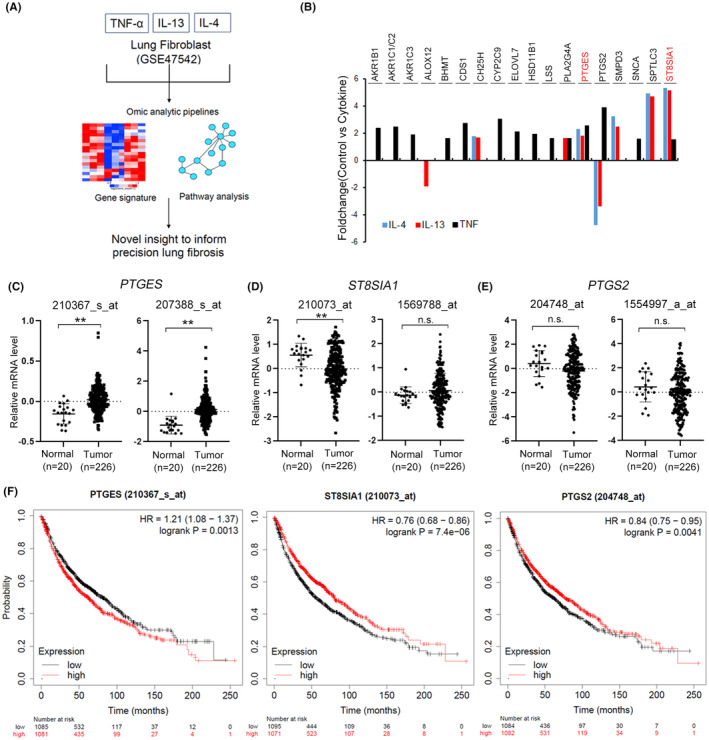
*PTGES* was upregulated in patients with lung cancer. (A) Schematic of the study guide in lung fibroblasts using GSE47542. (B) RNA levels of lipid metabolism genes relative to the control. (C–E) RNA levels of *PTGES*, *ST8SIA1* and *PTGS2* relative to the control. Expression levels of *PTGES* (C), *ST8SIA1* (D) and *PTGS2* (E) in patients with lung cancer (GSE31210). The data are represented as the mean ± SD; *p < 0.05; **p < 0.01; n.s., not significant. Student's *t*‐test was used to determine the significance. (F) *PTGES*, *ST8SIA1* and *PTGS2* expression levels for overall survival assessment using the Kaplan–Meier analysis (Kaplan–Meier plotter).

The *PTGES*, *ST8SIA1* and *PTGS2* mRNA expression was determined using the GSE profiles (IPF‐GSE2052, Figure S1) and datasets (lung cancer‐GSE31210, Figure 1C–E).

### 2.2 Cell culture

The MRC‐5 (14‐week gestation, lung fibroblast) and IMR‐90 (16‐week gestation, lung fibroblast) cell lines were maintained in α‐minimum essential (α‐MEM) medium (#11900024, Gibco™, USA) supplemented with 0.1 mM non‐essential amino acids, 1 mM sodium pyruvate, 10% fetal bovine serum (FBS) and 1% penicillin/streptomycin. The CL1‐0 (lung cancer) cells were cultured in RPMI‐1640 medium with 10% FBS and 1% penicillin/streptomycin, incubating them at 37°C in a humidified incubator under 5% CO_2_.

### 2.3 Recombinant protein treatment and conditioned medium

The MRC‐5 and IMR‐90 lung fibroblast cell lines were plated onto six‐well plates at a concentration of 3 × 10^5^ cells/well and incubated them overnight at 37°C. After incubation, the cells were treated with the recombinant protein IL‐4 (0–100 ng/mL), IL‐13 (0–20 ng/mL) and PTGES (0–50 ng/mL) for 144 h at 37°C. After 144 h, condition medium samples were collected and centrifuged at 1500 rpm and 4°C for 10 min to remove the cell debris. Lung fibroblast cells were treated with recombinant protein for 72, 96 and 144 h. After 144 h, α‐SMA was upregulated in fibroblast cells. Based on the upregulated α‐SMA expression to determine the recombinant protein treatment condition (concentrations and time). We referred to previous in vitro studies concerning the IL‐4 and IL‐13 concentrations used in this study.^42^, ^43^ All recombinant protein treatment experiment data are based on at least three independent replicates.

### 2.4 Lentivirus infection

Lung fibroblast cells were infected with pLVX‐IRES‐PTGES virus (8 μg/mL Polybrene) to express PTGES and with pLVX‐IRES‐Neo as a vector control. After 96 h of infection, cells were collected to confirm the PTGES expression using quantitative reverse transcription real‐time PCR (RT‐qPCR).

### 2.5 RNA extraction and RT‐qPCR

After viral infection, MRC‐5/Vector, MRC‐5/PTGES, IMR‐90/Vector and IMR‐90/PTGES were collected to confirm the corresponding expression using RT‐qPCR (SYBR system). The TRIzol® Reagent (ThermoFisher, #15596018) was used to extract total RNA. Next, PrimeScript™ RT Reagent Kit (Takara, #RR037A) was used to create complementary DNA (cDNA). Subsequently, target gene expression was assessed using real‐time PCR and the SYBR™ Green PCR Master Mix (Biosystems qPCRBIO SyGreen Mix Lo‐ROX). RT‐qPCR was performed using the appropriate primers listed in Table S3. All RT‐qPCR data are based on at least three independent replicates.

### 2.6 Western blotting

Proteins from the MRC‐5 and IMR‐90 cells were extracted using RIPA buffer and the protein concentrations were measured using the BCA protein assay kit (Pierce™ BCA Protein Assay Kit; Thermo Fisher, IL, USA). The proteins were loaded onto an SDS‐PAGE gel (10 and 15% based on target protein size) and, after separation, they were transferred onto a polyvinylidene difluoride membrane, which was blocked with 5% bovine serum albumin in PBST. The antibodies and conditions are listed in Table S3. The densitometric analysis of the protein bands was performed using image J and the target protein expression was normalized to the internal control (GAPDH or β‐actin).

### 2.7 Transwell migration/invasion assay

The cell migration assay was performed using the Transwell (Falcon™ HTS Multiwell Insert System) membrane filter inserted into 24‐well tissue culture plates (8 μm pore size, BD Biosciences, San Jose, CA, USA). After 144 h of treatment with the recombinant proteins IL‐4 (0–100 ng/mL), IL‐13 (0–20 ng/mL) and PTGES (0–50 ng/mL), the lung fibroblast cells were trypsinized, suspended in a serum‐free medium and seeded on the upper chamber of the Transwell filters. Next, the serum‐containing medium was added to the lower chamber and the set‐up was incubated for 48 h at 37°C. Next, the cells were fixed with 4% formaldehyde and stained with crystal violet. The non‐migrating cells were removed by wiping the upper side of the filter. Cell migration was determined using the Image J software. All invasion and migration data are based on at least three independent replicates.

### 2.8 Growth curve assay

After co‐culture with MRC‐5 (rPTGES treatment), the CL1‐0 cells were seeded at a concentration of 5000 cells/well onto a 96‐well plate for 24–72 h (incubated at 37°C under 5% CO_2_). A 3‐(4,5‐Dimethylthiazol‐2‐yl)‐2,5‐Diphenyltetrazolium Bromide (MTT) assay was used to determine the cell growth curve. Cell viability data are based on at least three independent replicates.

### 2.9 Animal model

Six‐week‐old and 200–250‐g male Sprague–Dawley rats (BioLASCO Co., Ltd., Taipei, Taiwan) were used for the in vivo study. For lung fibrosis formation, monocrotaline (MCT; 60 mg/kg) was administered by a single subcutaneous injection. After 28 d, the animals were euthanized and fibrosis was detected using Trichrome Stain (# ab150686, Abcam).

### 2.10 Immunohistochemistry

Immunohistochemical staining was performed according to the manufacturer's instructions. The primary antibodies and treatment conditions are summarized in Table S3. α‐SMA and PTGES expression was assessed as the intensity and percentage of positively stained cells using the HistoQuest software (TISSUEGNOSTICS). The expression scores were computed from the intensity (scale: 0–3.) × percentage of positively stained cells.

### 2.11 Statistical Analysis

All statistical analyses were performed using the SPSS 19.0 statistical package. A two‐tailed Student's *t*‐test was applied to identify significant differences between the two treatment groups and Tukey's post hoc test was performed to establish the levels of significance among the three treatment groups. *p* < 0.05 was considered statistically significant.

## 3 RESULTS

### 3.1 PTGES identification in lung fibroblast cells after cytokine treatment

To identify the lung fibrosis progression‐associated metabolic pathway, differentially expressed genes (1.5‐fold change) between the control and IL‐4‐, IL‐13‐ and TNF‐α‐treated cells (GSE47542) were analysed. To facilitate healing, fibroblasts should actively migrate to the site of injury.^44^ Therefore, we focused on analysing the Matrigel‐treated group (control Matrigel vs. cytokine Matrigel) (Figure 1A). By filtering the gene signature for the enzyme, our Ingenuity Pathway Analysis (IPA) of molecular and cellular functions identified lipid metabolism in lung fibroblast cells upon treatment with different cytokines (Table S1). Next, we focused on lipid metabolism. Similar to our prediction, previous studies have demonstrated PTGS2 (i.e. COX‐2) downregulation in lung myofibroblasts.^45^ During lipid synthesis, PTGES and ST8 alpha‐N‐acetyl‐neuraminide alpha‐2,8‐sialyltransferase 1 (ST8SIA1) expression was upregulated in IL‐4, IL‐13 and TNF‐α‐treated lung fibroblasts (Figure 1B). Next, *PTGES*, *ST8SIA1* and *PTGS2* expression levels were evaluated in patients with lung cancer (GSE31210) and IPF (GSE2052) using microarray datasets. As expected, *PTGES* was upregulated in lung cancer tumours and IPF‐affected lungs compared with healthy tissues (Figures 1C–E and S1). Moreover, high‐expression *PTGES* was associated with poor overall survival in patients with lung cancer (Figure 1F).

### 3.2 PTGES enhanced the migration and invasion abilities of lung fibroblast cells

**FIGURE 2:**
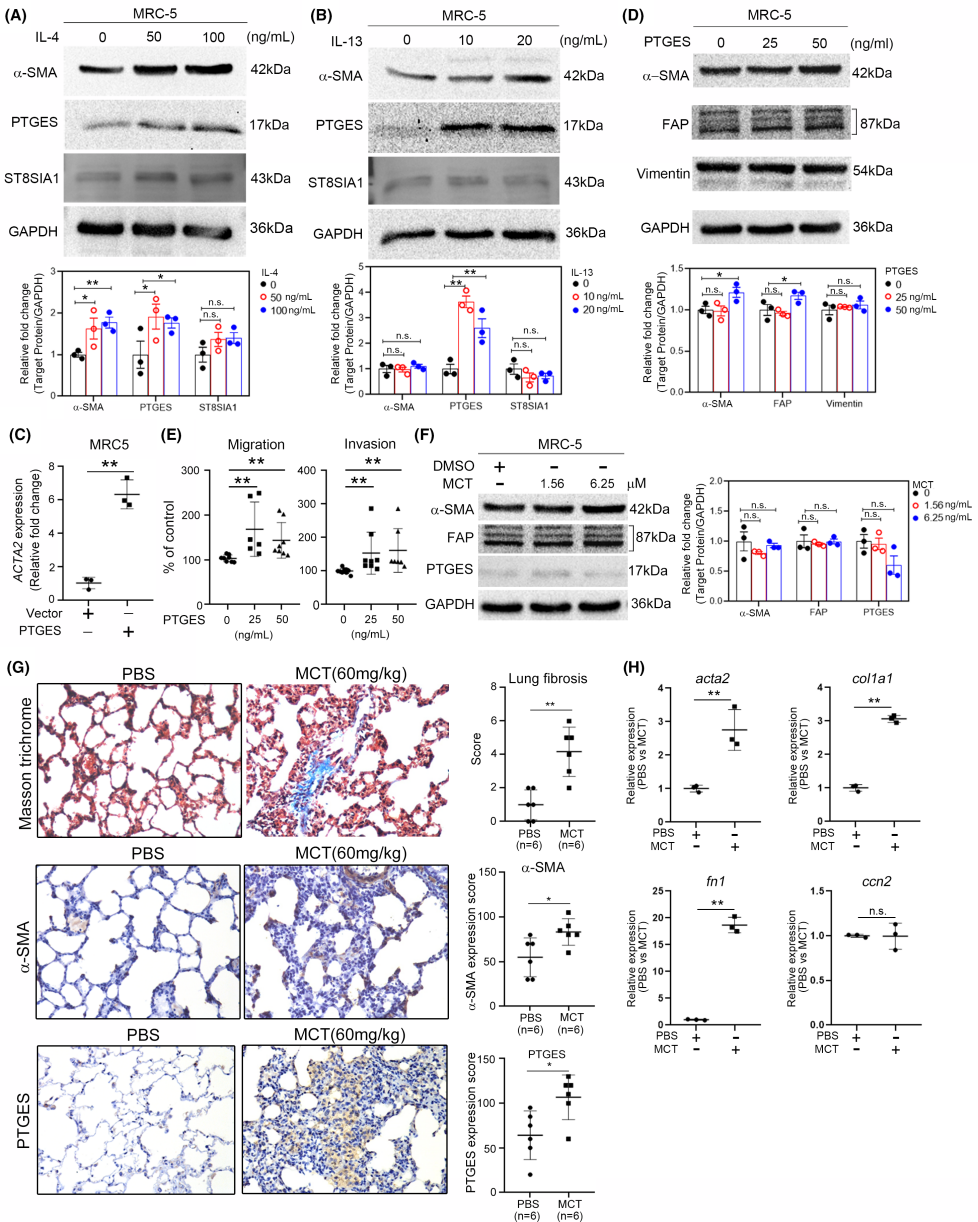
PTGES promotes migration and invasion abilities in MRC‐5 cells. IL‐4 and IL‐13 induce myofibroblast differentiation. MRC‐5 cells were treated with IL‐4 (A) or IL‐13 (B) for 144 h, and α‐SMA, PTGES and ST8SIA1 expression was analysed using western blotting. GAPDH was used as loading control. (C) *ACTA2* mRNA expression in PTGES‐overexpressing MRC‐5 cells compared with pLVX‐IRES‐Neo (vector control) (*n* = 3). MRC‐5 cells were treated with rPTGES for 144 h and α‐SMA expression was analysed using western blotting (D), while their migration/invasion ability (E) was assessed using Transwell chambers (*n* = 4). The results are displayed as the mean ± SD. Tukey's post hoc test was used to establish the significance after one‐way ANOVA. **p < 0.01. (F) Western blot analysis of α‐SMA, FAP and PTGES expression following monocrotaline (MCT) treatment in MRC‐5 cells. (G) Representative lung section images of Trichrome Stain staining, and α‐SMA and PTGES expression in PBS and MCT‐treated rats (*n* = 6). (H) RT‐qPCR analysis of *acta2*, *col1a1*, *fn1* and *ccn2* in the lung tissues of PBS and MCT‐treated rats (*n* = 3). The data represent the mean ± SD. Significance was determined using the Student's *t*‐test. *p < 0.05; **p < 0.01.

Next, we evaluated how cytokines affect α‐SMA (myofibroblast markers), PTGES and ST8SIA1 expression levels in MRC‐5 cells. Upregulated PTGESand α‐SMA expression was evaluated upon IL‐4 and IL‐13 treatments (Figure 2A,B, respectively). We further evaluated how IL‐4 or IL‐13 affect the migration and invasion abilities of the MRC‐5 cells and observed promotion in all cases (Figure S2A,B). Furthermore, *PTGES* was upregulated in lung tumours and was associated with poor overall survival in patients with lung cancer (Figure 1C,F). Therefore, we focused on PTGES in our further investigations. Next, we evaluated how PTGES affects myofibroblast differentiation. PTGES overexpression significantly upregulated Actin Alpha 2, Smooth Muscle (*ACTA2*) expression in MRC‐5 cells (Figures 2C and S2C). A similar result was observed upon rPTGES treatment, presenting α‐SMA and fibroblast activation protein alpha (FAP) upregulation (Figures 2D and S2D). Profibrotic marker collagen type I alpha 1 chain (*COCL1A*) and fibronectin 1 (*FN1*) were also upregulated in MRC‐5 and IMR‐90 cells upon rPTGES treatment (Figure S2E,F). We further evaluated how PTGES affected migration and invasion abilities and observed enhancement in MRC‐5 cells (Figures 2E and S2G).

Next, we evaluated PTGES expression levels in the case of lung fibrosis in rats. A previous study has demonstrated that monocrotaline (MCT) induces lung fibrosis.^46^ Our western blot analysis confirmed that α‐SMA andPTGES were upregulated in MRC‐5 cells upon MCT treatment (Figure 2F). In this study, MCT was subcutaneously injected into the rats and the induced lung fibrosis was observed. Next, α‐SMA and PTGES expression levels were evaluated in MCT‐treated rats and MCT‐induced upregulated α‐SMA and PTGES expression was detected (Figure 2G). Finally, RT‐qPCR was used to evaluate the following profibrotic marker expression in the lung tissue of MCT‐treated rats: *acta2*, *col1a1*, *fn1* and cellular communication network factor 2 (*ccn2* or *ctgf*), yielding *acta2*, *col1a1* and *fn1* upregulation (Figure 2H).

### 3.3 Myofibroblast‐stimulated tumour cell migration was PTGES‐dependent

**FIGURE 3:**
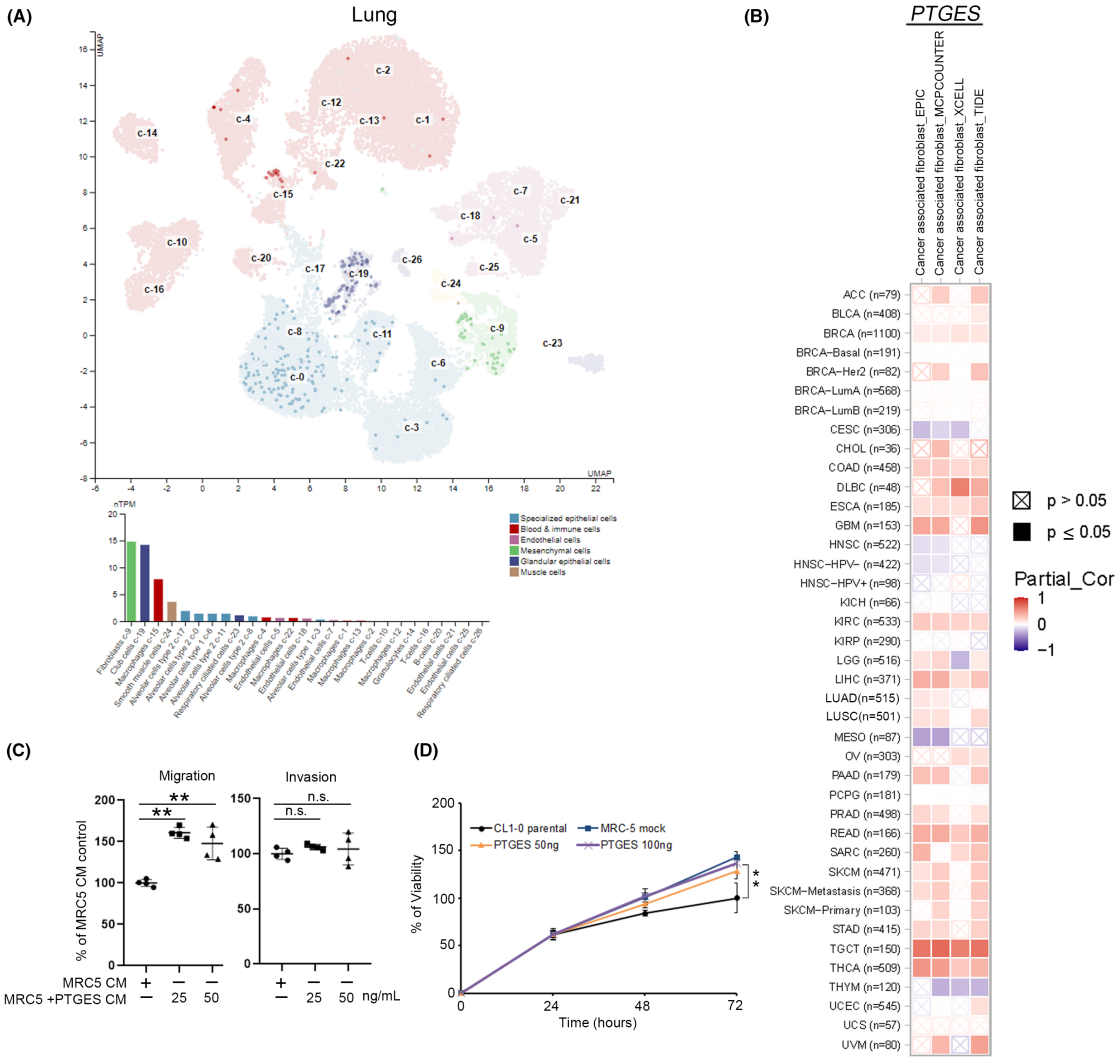
Correlation between *PTGES* and cancer‐associated fibroblasts. (A) Identified single‐cell type clusters in the lung tissue via single‐cell RNA sequencing represented in UMAP plots and bar graphs (HPA dataset). (B) Relationship between cancer‐associated fibroblasts and *PTGES* mRNA expression (TIMER). (C) The effect of conditioned medium of myofibroblasts (PTGES induces) in lung cancer cell migration. CL1‐0 cells were treated with a conditioned myofibroblast medium and their migration/invasion abilities were assessed using Transwell chambers (*n* = 4). (D) CL1‐0 cell treatment with MRC5‐conditioned medium for 72 h followed by cell viability analysis using MTT assay (*n* = 8). The data are represented as the means ± SD. Tukey's post hoc test was used to establish significances after one‐way ANOVA. **p < 0.01; n.s., not significant.

Transcriptome data were examined and the various cell types in the lung tissue were clarified using single‐cell RNA sequencing datasets of the Human Protein Atlas to support our findings. Uniform Manifold Approximation and Projection (UMAP) plots and bar graphs revealed that *PTGES* was more highly expressed in fibroblasts than in other cell types (Figure 3A). The relationship between *PTGES* and CAFs was further analysed in multiple cancers through different databases (TIMER), observing a significant association between them in multiple cancer types, including lung cancer (Figure 3B). Next, to investigate how PTGES‐induced myofibroblasts affect cancer progression, MRC‐5 fibroblast cells were treated with rPTGES; after 144 h, the conditioned medium (CM) was collected and used to treat CL1‐0 cells. The rPTGES‐derived CM increased CL1‐0 cell migration compared with the control (Figure 3C), although it did not affect viability (Figure 3D).

### 3.4 PTGES‐induced α‐SMA expression via HIF‐1α in lung fibroblast cells

**FIGURE 4:**
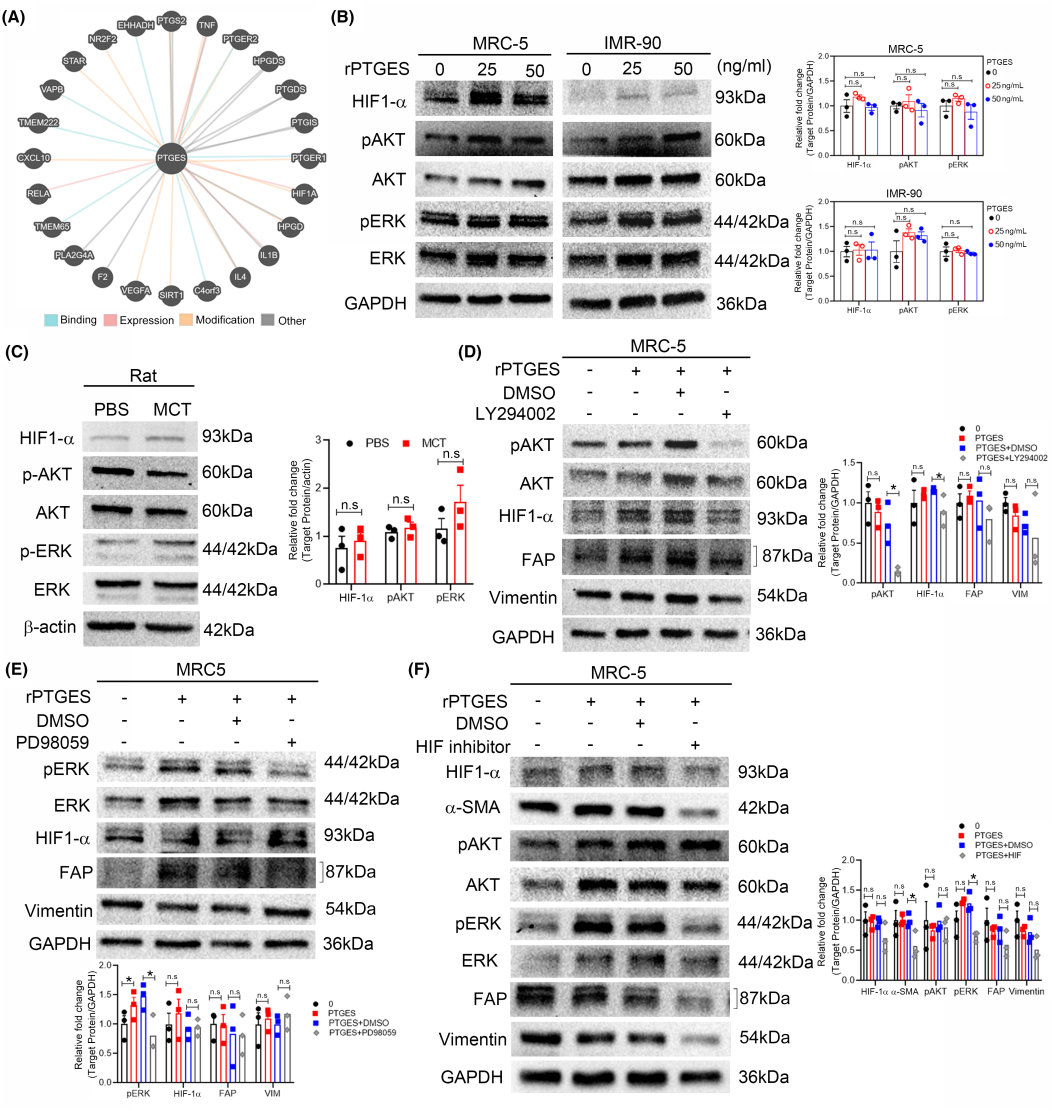
PTGES induced HIF‐1α expression in lung fibroblast cells. (A) Putative interaction targets were identified from Pathway Commons. (B) Western blot analysis of pAKT, AKT, HIF‐1α, pERK, ERK and GAPDH expression in MRC‐5 and IMR‐90 after rPTGES treatment. (C) Western blot results highlighting HIF‐1α, pAKT, AKT, pERK, ERK and β‐Actin expression in the MCT‐induced rat lung tissue. Western blot analysis of pAKT, AKT, HIF‐1α, pERK, ERK and GAPDH expression in MRC‐5 cells (PTGES pre‐treatment of 25 ng/mL) after treatment with (D) 10 μM of LY294002 or (E) 20 μM of PD98059. (F) Western blot analysis of HIF‐1α, α‐SMA, pAKT, AKT, pERK, ERK, FAP, Vimentin and GAPDH expression in MRC‐5 cells after 30 μM of HIF‐1α inhibitor treatment. The data represent the mean ± SD. Significance was determined using the Student's *t*‐test. *p < 0.05. n.s., not significant.

To identify the potential molecular targets of PTGES, we used the Pathway Commons database (https://www.pathwaycommons.org/). Our results indicated that PTGES interacted with hypoxia‐inducible factor 1 subunit alpha (HIF‐1α) (Figure 4A and Table S2). A previous study has described that MCT induces HIF‐1α expression in rats. Moreover, PDGF induces HIF‐1α expression via the AKT/ERK pathway in human pulmonary artery smooth muscle cells.^47^ We then determined whether PTGES activates HIF‐1α, AKT and ERK expression. Our western blotting analysis demonstrated that HIF‐1α expression and AKT/ERK phosphorylation were consistently upregulated after rPTGES treatment in MRC‐5 and IMR‐90 cells (Figure 4B). Furthermore, HIF‐1α and ERK phosphorylation were also upregulated in the lung tissue of MCT‐treated rats (Figure 4C). Next, we examined whether PTGES mediates HIF‐1α expression through AKT/ERK in lung fibroblast cells using the PI3‐kinase inhibitor LY294002, yielding reduced HIF‐1αexpression in rPTGES‐treated lung fibroblast cells (Figure 4D). However, PD98059 (MEK/ERK inhibitor) did not significantly inhibit HIF‐1α expression upon rPTGES treatment (Figure 4E). Moreover, the AKT/ERK pathway was reportedly activated under hypoxic conditions.^47^ Therefore, we explored whether HIF‐1α could induce AKT/ERK phosphorylation in lung fibroblast cells. CoCl_2_ treatment led to significant AKT/ERK phosphorylation in the MRC‐5 cells (Figure S3). We further explored the HIF‐1α modulatory effect on α‐SMA expression in lung fibroblasts and observed significant α‐SMA downregulating in MRC‐5 upon HIF‐1α inhibitor treatment. Moreover, the HIF‐1α inhibitor treatment reduced ERK phosphorylation in rPTGES‐treated lung fibroblast cells, although it did not significantly reduce AKT phosphorylation following rPTGES treatment (Figure 4F). These data indicate that PTGES mediates HIF‐1α expression through AKT activation.

### 3.5 PTGES promoted glycolysis through HIF‐1α in fibroblasts

**FIGURE 5:**
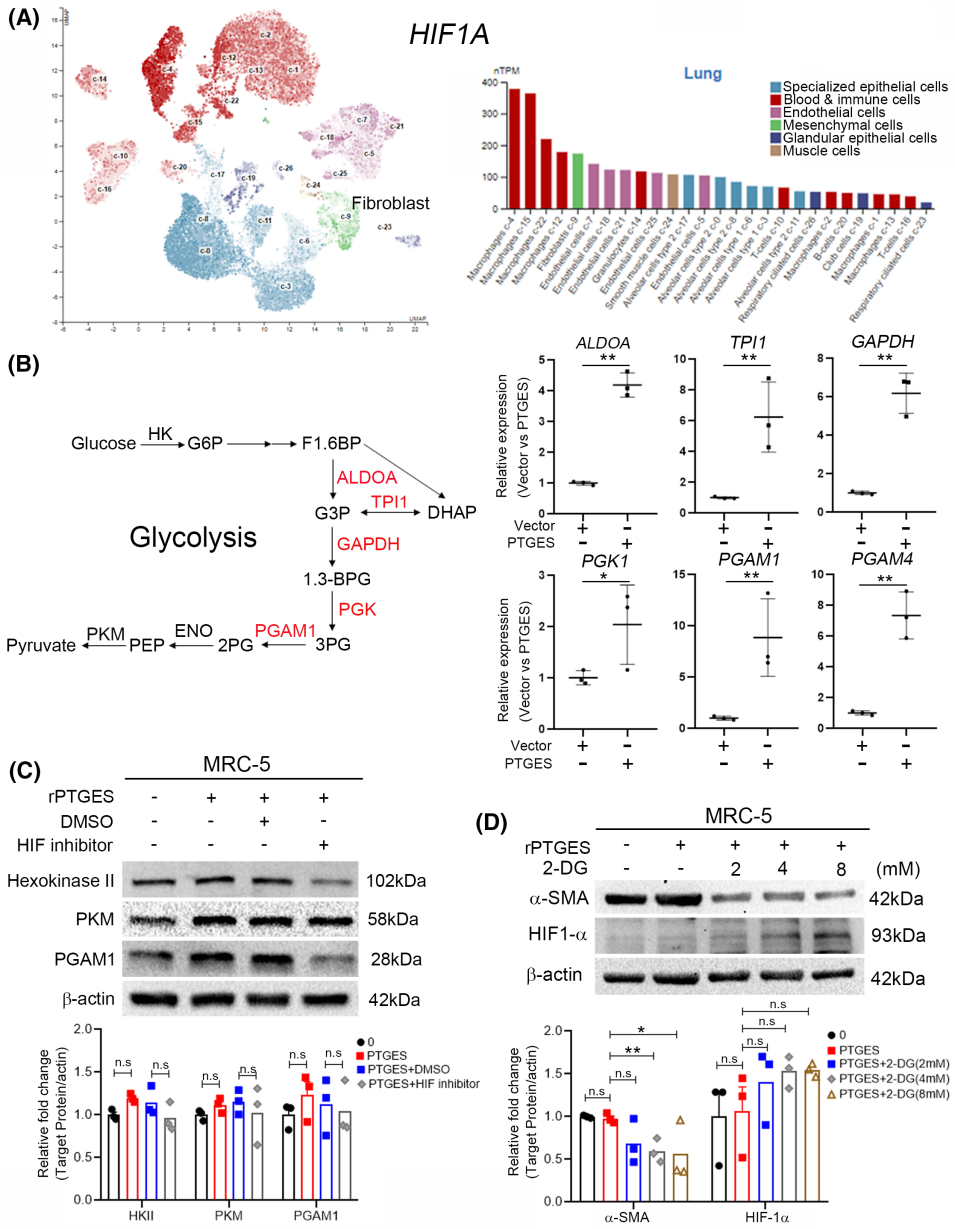
PTGES induced glycolysis via HIF‐1α in lung fibroblast cells. (A) As demonstrated by the UMAP plots and bar graphs (HPA dataset), single‐cell RNA sequencing was used to identify *HIF1A* expression single‐cell type clusters in the lung tissue. (B) Left, Schematic diagram of glycolysis pathway. Right, RT‐qPCR analysis of *ALDOA*, *TPI1*, *GAPDH*, *PGK1*, *PGAM1* and *PGAM4* in PTGES‐overexpressing and vector control cells (*n* = 3). The data are represented as mean ± SD, **p < 0.01; *p < 0.05. The gene expression values are normalized to *ACTB*. (C) Western blot analysis of HK2, PKM, PGAM1 and β‐Actin expression in MRC‐5 cells (PTGES pre‐treatment of 25 ng/mL) after treatment with 30 μM of HIF‐1α inhibitor. (D) Western blot analysis of α‐SMA, HIF‐1α and β‐Actin expression in MRC‐5 cells after 2‐deoxy‐D‐glucose (2‐DG) treatment. β‐Actin was used as loading control. The data represent the mean ± SD. Significance was determined using the Student's *t*‐test. **p < 0.01; *p < 0.05. n.s., not significant.

In our study, HIF‐1α is central to myofibroblast differentiation. We further evaluated *HIF1A* expression using single‐cell RNA sequencing datasets (HPA) to analyse transcriptomic data and identify the different cell types in the lung tissue. Our UMAP plots and bar graphs indicated similar results, that is, *HIF1A* was upregulated in macrophages and fibroblasts compared with that in other cell types (Figure 5A). In addition, the HIF‐1α inhibitor reduced GAPDH expression in rPTGES‐treated MRC5 cells (Figure 4F). HIF‐1α is a reported glycolysis pathway‐mediating transcription factor in several cell types, including fibroblast.^48^ Next, we further explored the possibility of whether PTGES could induce glycolysis and observed significantly upregulated glycolysis biosynthesis enzymes upon PTGES overexpression in the MRC‐5 cells as follows^1^: aldolase, fructose‐bisphosphate A (*ALDOA*)^2^; triosephosphate isomerase 1 (*TPI1*)^3^; glyceraldehyde‐3‐phosphate dehydrogenase (*GAPDH*)^4^; phosphoglycerate kinase 1(*PGK1*)^5^; phosphoglycerate mutase 1(*PGAM1*)^6^; phosphoglycerate mutase family member 4 (*PGAM4*) (Figure 5B). Moreover, a similar result was observed following rPTGES treatment, yielding upregulated glycolysis enzymes (Hexokinase 2, HK2; Pyruvate Kinase M1/2, PKM; PGAM1) in the MRC‐5 cells (Figure 5C). Similar results were observed in the fibrotic lung with significantly upregulated glycolysis biosynthesis enzymes in the lung tissue of MCT‐treated rats (Figure S4). Furthermore, HIF‐1α inhibitor treatment reduced the HK2 and PGAM1 levels in rPTGES‐treated lung fibroblast cells (Figure 5C), indicating potential PTGES‐HIF‐1α involvement in the glycolysis pathway. Moreover, we investigated whether the glycolysis pathway was involved in myofibroblast formation. Treatment with the glycolysis inhibitor 2‐deoxyglucose (2‐DG) reduced α‐SMA expression in rPTGES‐treated lung fibroblast cells (Figure 5D). These data suggest that PTGES enhanced the glycolysis pathway via HIF‐1α, leading to myofibroblast differentiation.

### 3.6 PTGES targeting inhibited myofibroblast differentiation

**FIGURE 6:**
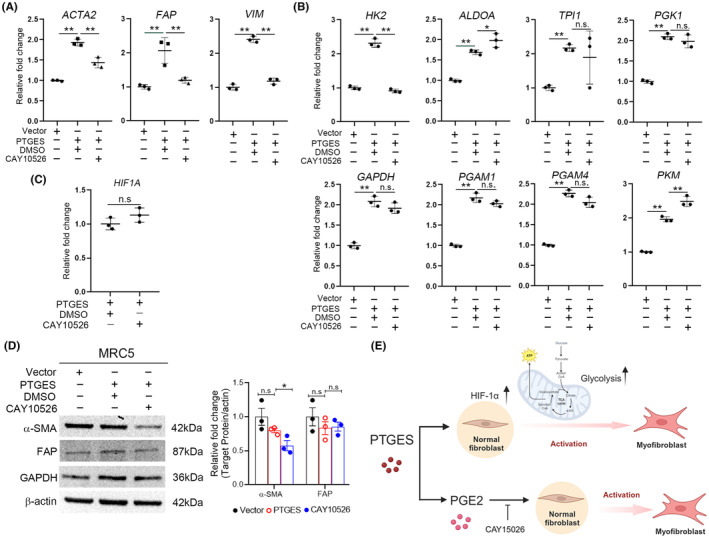
Targeting PTGES/PGE_2_ signalling inhibits myofibroblast differentiation in MRC‐5 cells. PTGES‐overexpressing cells were treated with 5 μM of CAY10526 for 48 h, followed by the expression analysis of (A) *ACTA2*, *FAP*, *VIM*, (B) glycolysis pathway genes, and (C) *HIF1A* using RT‐qPCR (*n* = 3). The data are represented as the mean ± SD. *p < 0.05; **p < 0.01; n.s., not significant. Tukey's post hoc test was used to establish significances after one‐way anova. **p < 0.01; n.s., not significant. (D) Western blot results indicating the protein levels of α‐SMA, FAP, GAPDH and β‐Actin in the lung tissue with or without MCT treatment. β‐Actin was used as loading control. The data represent the mean ± SD. Significance was determined using the Student's *t*‐test. *p < 0.05. (E) Model of the roles of PTGES in lung fibroblast. PTGES induced the upregulation of HIF‐1α expression and the glycolysis pathway, leading to myofibroblast differentiation.

PTGES is an enzyme responsible for catalyzing prostaglandin H2 (PGH_2_) conversion into a prostaglandin E2 (PGE_2_) metabolite. We explored the PTGES/PGE_2_ signalling involvement in myofibroblast differentiation through PTGES inhibitor CAY10526 treatment, leading to *ACTA2*, *FAP* and *VIM* downregulation in PTGES‐overexpressing cells (Figure 6A). Next, we investigated whether the PTGES/PGE_2_ signalling mediated the glycolysis pathway in PTGES‐overexpressing cells and observed this pathway mediated myofibroblast differentiation without significantly impacting the glycolysis pathway (Figure 6B). We further evaluated the PTGES/PGE_2_ signalling that affects HIF‐1α expression in PTGES‐overexpressing cells. The CAY10526 treatment did not significantly reduce *HIF1A* expression in the MTC5/PTGES cells (Figure 6C). We obtained similar results related to the protein levels, demonstrating downregulated α‐SMA expression in PTGES‐overexpressing cells upon the CAY10526 treatment (Figure 6D). Taken together, our results suggest that PTGES mediates AKT phosphorylation, leading to upregulated HIF‐1α‐dependent glycolysis and contributing to myofibroblast differentiation (Figure 6E).

### 3.7 PTGES modulated lipogenic enzyme expression in lung fibroblast cells

In our study, we revealed PTGES functions related to lipid metabolism in lung fibroblast cells upon treatment with cytokines (Table S1). We evaluated whether PTGES mediates lipogenic enzymes in lung fibroblast cells by analysing the expression of fatty acid synthase (*FASN*), stearoyl‐CoA desaturase (*SCD*), acetyl‐CoA carboxylase alpha (*ACACA*), ELOVL fatty acid elongase 6 (*ELOVL6*) and diacylglycerol O‐acyltransferase 2 (*DGAT2*) using RT‐qPCR. PTGES overexpression significantly increased the expression of *FASN*, *SCD*, *ACACA*, *ELOVL6* and *DGAT2* in PTGES‐overexpressing MRC‐5 cells (Figure S5).

## 4 DISCUSSION

In this study, we identified PTGES via differential expression analysis in lung fibroblast cells after treatment with selected cytokines (GSE47542).^43^ We speculated that *PTGES* expression was upregulated in patients with IPF and lung cancer. We observed that PTGES enhanced the migration and invasion abilities and myofibroblast marker expressions in lung fibroblast cells. Furthermore, PTGES was also upregulated in the fibrotic lung tissue of rats. Taken together, these results suggest that PTGES may play an important role in lung fibroblast function. Specifically, our study first indicated that the lipid synthesis protein PTGES regulated lung fibroblast function, both in vitro and in vivo via a de novo pathway, underpinning that PTGES‐mediated myofibroblast differentiation induced HIF‐1α expression via AKT‐signalling in lung fibroblast cells.

PGE_2_ synthases comprise the cytosolic PGE synthase (cPGES) and two membrane‐bound PGE synthases, PTGES and mPGES‐2, of which cPGES and mPGES‐2 are constitutive enzymes, whereas PTGES is inducible.^49^ PGE_2_ induces normal human lung fibroblasts to undergo more apoptotic signals that would be delivered by the Fas ligand. The PGE_2_ active EP2/EP4 signalling induces fibroblast apoptosis and reduces AKT activity.^50^ However, fibroblasts of patients with IPF are resistant to the proapoptotic effect of PGE_2_.^50^ Moreover, PGE_2_ could inhibit TGF‐β‐induced myofibroblast differentiation and limit collagen secretion.^51^ In contrast, PGE_2_ exerts profibotic effects via cyclin D expression enhancement to promote fibroblast proliferation.^52^ In this study, rPTGES‐induced myofibroblasts promoted the migration ability of lung cancer cells. Treatment with a PTGES inhibitor (CAY10526, inhibiting PGE_2_ production) reduced *ACTA2* expression in PTGES‐overexpressing cells. However, the PTGES/PGE_2_ signalling did not affect HIF‐1α signalling in mediating the glycolysis pathway in the case of PTGES overexpression. We detected the upregulation of PTGES and its corresponding mRNA levels in lung fibroblast cells after treatment with cytokines, suggesting that PTGES not only catalysed endoperoxide PGH_2_ conversion to PGE_2_ but also possessed undefined, non‐enzymatic functions. PTGES induced α‐SMA and FAP expression and increased lung fibroblast cell migration and invasion abilities.

In this study, we found that PTGES activated the AKT and ERK signalling pathways in lung fibroblast cells. PTGES and pERK expression were upregulated in the fibrotic lungs of rats after MCT treatment. Similar to our results, Cheng et al. also demonstrated that MCT induced lung fibrosis and increased HIF‐1α expression in rats, while MEK/ERK and PI3‐kinase inhibitor treatments (i.e. PD98059 and LY294002, respectively) significantly reduced HIF‐1α expression in PASMCs.^47^ MAPK/ERK is an important pathway regulator of cellular processes associated with fibrogenesis, such as growth, proliferation and survival. In human fibrotic lung samples, the MAPK/ERK pathway is reportedly activated.^53^, ^54^ Treated with MEK inhibitor (ARRY‐142886) in TGF‐α‐induced lung fibrosis mice inhibited lung cell proliferation and protected lung function change.^55^ Moreover, the PI3K/AKT pathway similarly affects lung fibrosis, AKT increases α‐SMA expression in fibrotic lungs and targets AKT to prevent fibroblast transition into myofibroblasts.^56^ In a bleomycin‐induced cell model, PI3K/AKT and HIF‐1α were upregulated, regulating fibroblast proliferation and collagen production. Treatment with a PI3K/AKT pathway inhibitor (LY294002 and wortmannin) reportedly reduced HIF‐1α expression in bleomycin‐treated fibroblast cells.^57^ In our study, treatment with a PI3‐kinase inhibitor significantly reduced HIF‐1α expression in rPTGES‐treated lung fibroblast cells. However, HIF‐1α did not respond to ERK inhibitors in lung fibroblast MRC‐5 cells.

HIF‐1α is an important component in fibrotic tissues, enhancing myofibroblast differentiation and fibrotic disease development, including those in the lungs.^58^, ^59^ When hypoxia is advanced in a microenvironment, it promotes HIF‐1α‐dependent myofibroblast differentiation.^59^ HIF‐1α regulates metabolic processes, providing an important pathobiological step in fibrotic development. TGF‐β is a metabolic regulator that mediates the glycolysis pathway by stabilizing HIF‐1α, leading to pyruvate dehydrogenase kinase 1 (PDK1) activation.^60^ Lactate accumulation could also be observed in patients with IPF and during TGF‐β‐induced myofibroblast differentiation. Inhibition of the TGF‐β/HIF‐1α/PDK1 axis via deletion of HIF‐1α expression or targeting PDK1 by drug‐reduced pulmonary fibrosis.^59^, ^61^ In our study, treatment with a HIF‐1α inhibitor significantly reduced α‐SMA expression in rPTGES‐treated MRC‐5 cells. PTGES may provide another pathway to induce myofibroblast differentiation. Moreover, myofibroblast transition to a lipofibroblast (as a precursor cell for the myofibroblast) results in fibrosis resolutions.^62^ Reverse lung fibrosis by metformin reportedly induced lipogenic differentiation and an increase in lipid droplet accumulation.^35^ PTGES function was categorized as lipid metabolism‐related and its overexpression increased lipogenesis markers in fibroblasts. We cannot exclude the possibility that other pathways would be involved in myofibroblast differentiation. Finally, this study demonstrates that PTGES induces myofibroblast differentiation.

## 5 CONCLUSION

In this study, we identified PTGES as a myofibroblast differentiation‐associated modulatory regulator. Significantly, we discovered a novel myofibroblast differentiation‐associated PTGES/HIF‐1α and PTGES/AKT/ERK pathway. Taken together, our data suggest that PTGES is a propitious fibrosis therapeutic target and a novel biomarker in lung fibrosis.

## AUTHOR CONTRIBUTIONS

**Min‐Hsi Lin:** Data curation (supporting); validation (supporting). **Yi‐Chen Lee:** Resources (supporting); validation (supporting). **Jia‐Bin Liao:** Methodology (supporting); validation (supporting). **Chih‐Yu Chou:** Data curation (lead); formal analysis (supporting). **Yi‐Fang Yang:** Data curation (lead); funding acquisition (lead); investigation (lead); project administration (lead); writing – original draft (lead); writing – review and editing (lead).

## CONFLICT OF INTEREST STATEMENT

The authors declare that they have no conflict of interest.

## Supporting information


Appendix S1.

## Data Availability

The data presented in this study are available upon request from the corresponding author.
