# Supplementary material for: PTGES is involved in myofibroblast differentiation via HIF‐1α‐dependent glycolysis pathway
Source: J Cell Mol Med. 2024 Oct 17;28(20):e70157. doi: 10.1111/jcmm.70157 (PMC11484478; doi:10.1111/jcmm.70157)
Supplement: Supplementary file 1 — Appendix S1. [file JCMM-28-e70157-s001.docx]

**PTGES is involved in myofibroblast differentiation via HIF-1α-dependent glycolysis pathway**

Min-Hsi Lin^1^, Yi-Chen Lee^2^, Jia-Bin Liao^3^, Chih-Yu Chou ^4^ and Yi-Fang Yang^4*^

https://orcid.org/0000-0002-3622-0609 (Min-Hsi Lin)

https://orcid.org/0000-0002-4889-095X (Yi-Chen Lee)

https://orcid.org/0000-0001-7425-3156 (Yi-Fang Yang)

^1^Division of Chest Medicine, Kaohsiung Veterans General Hospital, Kaohsiung, Taiwan

^2^ Department of Anatomy, School of Medicine, College of Medicine, Kaohsiung Medical University, Kaohsiung, Taiwan

^3^Department of Pathology and Laboratory Medicine, Kaohsiung Veterans General Hospital, Kaohsiung, Taiwan

^4^Department of Medical Education and Research, Kaohsiung Veterans General Hospital, Kaohsiung, Taiwan

***Corresponding Author:**

Yi-Fang Yang, PhD

Department of Medical Education and Research, Kaohsiung Veterans General

Hospital, No. 386, Dajhong 1st Rd., Zuoying Dist., Kaohsiung City 813414, Taiwan. Phone: 886-7-342-2121 # 71592; Fax: 886-7-342-2288; E-mail: yvonne845040@gmail.com

Inventory of all Supplemental Information

Supplemental Data

Figure S1 Related to Figure1

Figure S2 Related to Figure2

Figure S3 Related to Figure4

Figure S4 Related to Figure5

Figure S5

Supplementary Tables

Supplementary Table 1 Related to Figure 1

Supplementary Table 2 Related to Figure 4

Supplementary Table 3 Related to Key Resources Table

**Supplementary Figure 1**


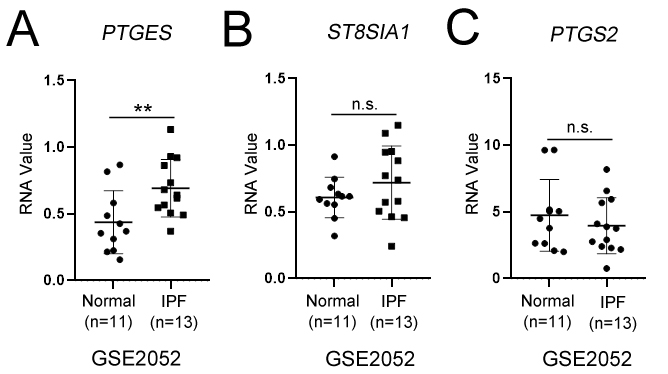


**Supplementary Figure S1.** RNA expression levels of *PTGES* (A), *ST8SIA1*(B), and *PTGS2* (C) in lung tissues of IPF patients (GSE2052). The Student *t*-test was used to determine the significance. Data are presented as the means ± SD; ***p* < 0.01. n.s, not significant.

**Supplementary Figure 2**


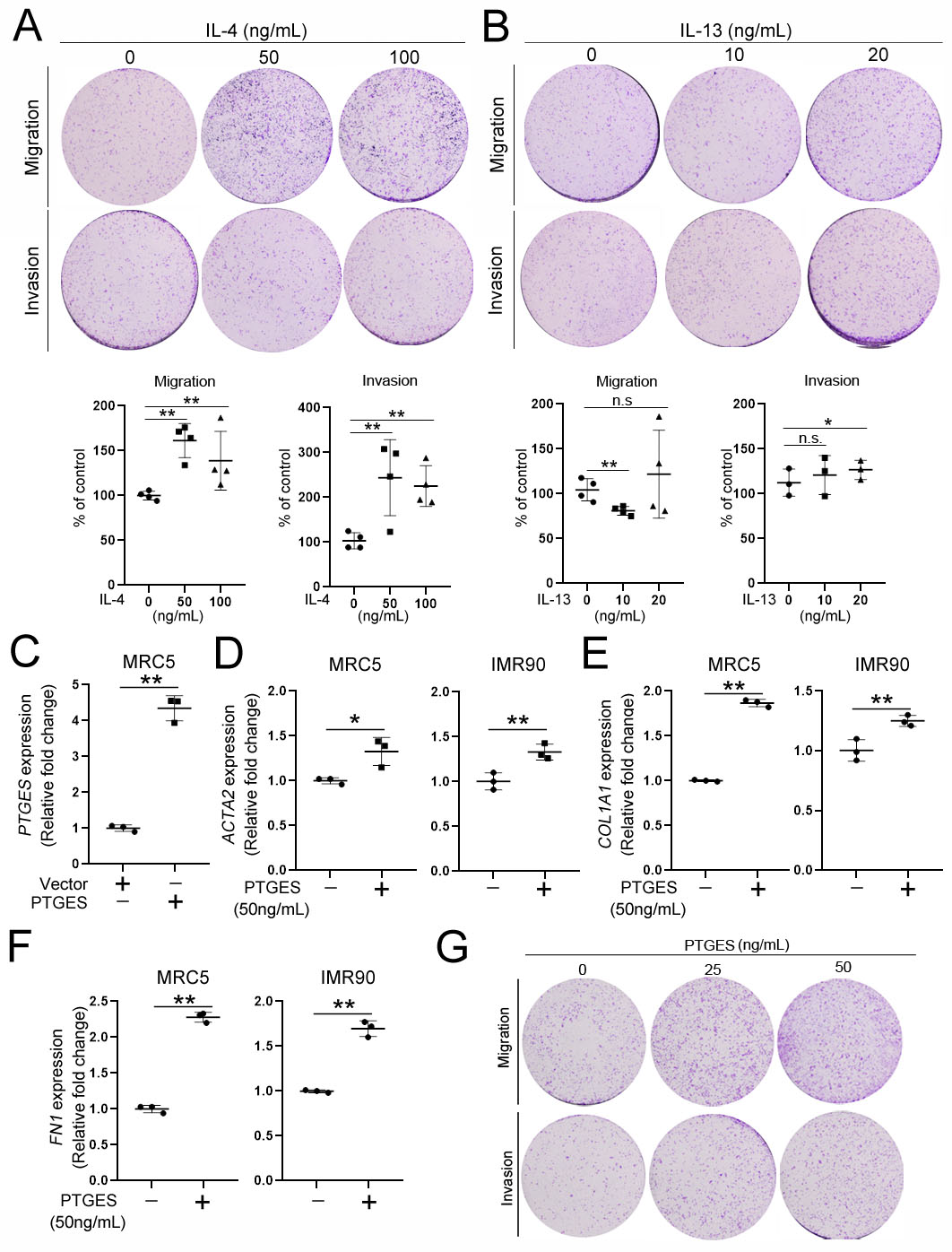


**Supplementary Figure S2.** Interleukin (IL)-4 and IL-13 promote migration and invasion abilities in lung fibroblast cells. MRC-5 cells were treated with IL-4 (A) or IL-13 (B) for 144 h, while the migration/invasion ability was assessed using transwell chambers (n=4). (C) RT-qPCR analysis of *PTGES* expression in MRC5 after virus infection. RT-qPCR analysis of *ACTA2* (D)*, COL1A1* (E)*,* and *FN1* (F) expression in MRC5 and IMR-90 after treatment with PTGES recombinant protein (n=3). Data were presented as the mean±SD, Values for gene expression are normalized to *GAPDH*. Tukey's *post hoc* test is used to establish significance after one-way ANOVA in (A-B) and the Student *t*-test was used to determine the significance in (C-F). **p* < 0.05; ***p* < 0.01. n.s, not significant. (G) Representative images of the migration/invasion assays of MRC5 cells treated with rPTGES for 144 h.


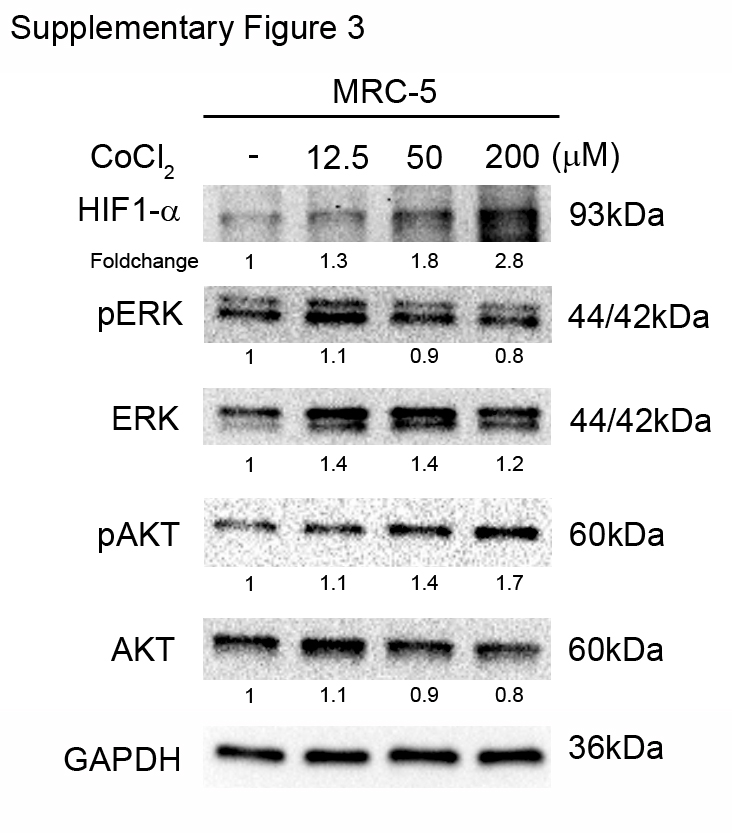


**Supplementary Figure S3.** MRC-5 cells were treated with CoCl_2_ (as HIF-1α inducer) for 24 h and analyzed by western blot.


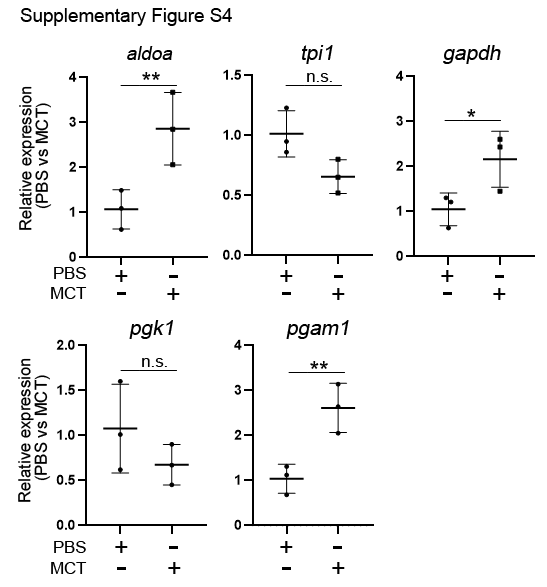


**Supplementary Figure S4.** Glycolysis pathway upregulated in lung tissues of PBS and MCT-treated rats. RT-qPCR analysis of *aldoa,* *tpi1*, *gapdh*, *pgk1*, and *pgam1* in MCT group and PBS group lung tissues (n=3). Data were presented as the mean±SD, ***p*<0.01; **p*<0.05. n.s, not significant. Values for gene expression are normalized to *actb*.


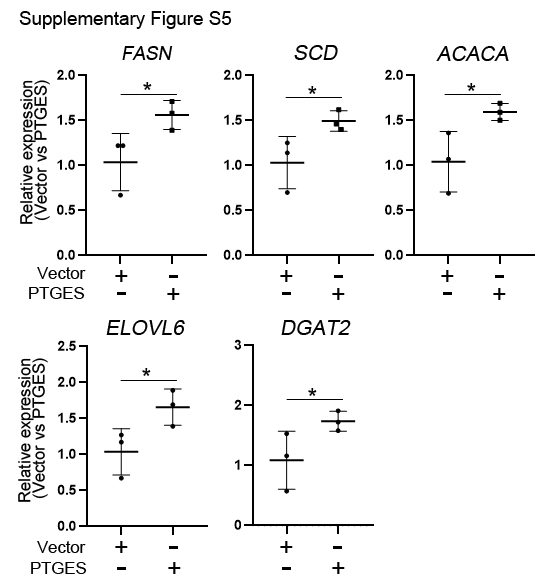


**Supplementary Figure S5.** PTGES drives lipogenic marker upregulation in lung fibroblast cells. RT-qPCR analysis of *FNSN,* *SCD*, *ACACA*, *ELOVL6*, and *DGAT2* in MCR-5/PTGES and MRC/5 vector cells (n=3). Data were presented as the mean±SD. The Student t-test was used to determine the significance. **p*<0.05. Values for gene expression are normalized to *ACTB*.

Supplementary Table S1. Molecular and Cellular Functions of IL-4, IL-13, and TNF-α

| Molecular and Cellular Functions of IL-4 | | | |
| --- | --- | --- | --- |
| Name | *p*-value range | Molecules | |
| Lipid Metabolism | 1.44E-02 - 4.21E-06 | 8 | |
| Small Molecule Biochemistry | 1.44E-02 - 4.21E-06 | 15 | |
| Molecular Transport | 1.44E-02 - 5.03E-05 | 6 | |
| Carbohydrate Metabolism | 1.19E-02 - 2.12E-04 | 5 | |
| Drug Metabolism | 8.42E-03 - 2.91E-04 | 6 | |
| Molecular and Cellular Functions of IL-13 | | | |
| Name | *p*-value range | | Molecules |
| Lipid Metabolism | 1.10E-02 - 5.58E-06 | | 9 |
| Small Molecule Biochemistry | 1.10E-02 - 5.58E-06 | | 16 |
| Cell-To-Cell Signaling and Interaction | 1.10E-02 - 1.86E-05 | | 10 |
| Nucleic Acid Metabolism | 1.10E-02 - 3.31E-05 | | 7 |
| Molecular Transport | 9.67E-03 - 4.30E-05 | | 8 |
| Molecular and Cellular Functions of TNF-α | | | |
| Name | *p*-value range | | Molecules |
| Lipid Metabolism | 6.61E-03 - 1.39E-07 | | 22 |
| Small Molecule Biochemistry | 6.61E-03 - 1.39E-07 | | 33 |
| Amino Acid Metabolism | 4.72E-03 - 7.84E-07 | | 10 |
| Molecular Transport | 6.33E-03 - 1.00E-05 | | 17 |
| Carbohydrate Metabolism | 6.61E-03 - 1.54E-05 | | 16 |

Supplementary Table S2. List of visualizing interactions between PTGES and candidate target genes.

| Candidate target | Interaction types |
| --- | --- |
| C4orf3 | interacts with PTGES |
| EHHADH | interacts with PTGES |
| HPGDS | catalysis |
| IL1B | controls expression of PTGES |
| PTGDS | catalysis |
| PTGER1 | interacts with PTGES |
| PTGER2 | interacts with PTGES |
| F2 | catalysis |
| HPGD | catalysis |
| HPGDS | catalysis |
| IL1B | catalysis |
| IL4 | catalysis |
| PLA2G4A | catalysis |
| PTGDS | catalysis |
| PTGIS | catalysis |
| PTGS2 | catalysis |
| TNF | catalysis |
| HIF1A | PTGES controls expression |
| CXCL10 | PTGES controls state change |
| HIF1A | PTGES controls state change |
| HPGD | PTGES controls state change |
| IL4 | PTGES controls state change |
| NR2F2 | PTGES controls state change |
| PTGER1 | PTGES controls state change |
| PTGER2 | PTGES controls state change |
| PTGS2 | PTGES controls state change |
| SIRT1 | PTGES controls state change |
| STAR | PTGES controls state change |
| TNF | PTGES controls state change |
| VEGFA | PTGES controls state change |
| PTGIS | catalysis |
| PTGS2 | catalysis |
| RELA | controls expression of PTGES |
| TMEM222 | interacts with PTGES |
| TMEM65 | interacts with PTGES |
| TNF | controls expression of PTGES |
| VAPB | interacts with PTGES |

Supplementary Table S3. Key resources table

| **Antibody** | **Source, catalog number** | **Application** | **Dilution** |
| --- | --- | --- | --- |
| α-SMA | Cell Signal, #19245s | Western blot | 1:1000,1:200* |
| Vimentin | GeneTex, GTX100619 | Western blot | 1:5000 |
| FAP | Abcam, ab28244 | Western blot | 1:1000 |
| PTGES | Invitrogen, PA5-60916 | Western blot, IHC* | 1:1000,1:50* |
| ST8SIA1 | Elabscience, E-AB-68025 | Western blot | 1:1000 |
| GAPDH | GeneTex, GTX100118 | Western blot | 1:5000 |
| HIF-1α | GeneTex, GTX127309 | Western blot | 1:2000 |
| pAKT(Ser473) | Cell Signal, #4060 | Western blot | 1:1000 |
| AKT(pan) | Cell Signal, #4691 | Western blot | 1:1000 |
| pERK1/2 | Cell Signal, #9101 | Western blot | 1:1000 |
| ERK1/2 | Cell Signal, #9102 | Western blot | 1:1000 |
| Hexokinase II | GeneTex, GTX111525 | Western blot | 1:1000 |
| PKM | GeneTex, GTX107977 | Western blot | 1:1000 |
| PGAM1 | Epitomics, 5787-s | Western blot | 1:1000 |
| β-actin | Sigma, A5441 | Western blot | 1:5000 |
| Abbreviations: IHC: immunohistochemistry | | | |
| **Reagent** | **Source, catalog number** | **Function** | **Concentration** |
| Monocrotaline (MCT) | Cayman Chemical, Item No. 16666 | animal model of pulmonary  hypertension. | 60mg/mL |
| Recombinant Human Interleukin 4 (IL–4) protein | Gibco, PHC0044 |  | 50-100ng/mL |
| **Continued** |  |  |  |
| Recombinant Human Interleukin–13 (IL–13) protein | Gibco, PHC0134 |  | 10-20ng/mL |
| PTGES (Human) Recombinant  Protein (P01) | Abnova, H00009536-P01 | - | 25-50ng/mL |
| LY294002 | Sigma-Aldrich, L9908 | PI3K Inhibitor | 10 μM |
| PD-98059 | Sigma-Aldrich, P215 | MAPK Inhibitor | 20 μM |
| Hypoxia-inducible factor-1α inhibitor | Santa Cruz Biotechnology, sc-205346 | Hypoxia-inducible factor-1α inhibitor | 30 μM |
| 2-Deoxy-D-glucose | Santa Cruz Biotechnology, sc-202010 | inhibitor of GPI | 2-8 mM |
| CAY10526 | Santa Cruz Biotechnology, sc-223868 | inhibitor of PTGES inhibits PGE_2_ production | 5 μM |
| **Critical Commercial Assays** | | | |
| Trichrome Stain (Connective Tissue Stain) | Abcam,# ab150686 | fibrosis |  |
| **Primer Sequence** | | | |
| **Gene** | **Sequence** | | |
| **Human** |  | | |
| *PTGES*- forward | CAG TAT TGC AGG AGC GAC CC | | |
| *PTGES*- reverse | GTG CAT CCA GGC GAC AAA AG | | |
| *ACTA2*- forward | CCA ACT GGG ACG ACA TGG AA | | |
| *ACTA2*- reverse | ATT TTC TCC CGG TTG GCC TT | | |
| *FAP-* forward | AGA ACC ATG CTT TGG AGA TAC T | | |
| *FAP-* reverse | TTT ACT CCC AAC AGG CGA CC | | |
| *VIM_* forward | GCT AAC CAA CGA CAA AGC CC | | |
| *VIM_* reverse | GAT TGC AGG GTG TTT TCG GC | | |
| *ALDOA*- forward | CAA ATC CAA GGG CGG TGT TG | | |
| *ALDOA*- reverse | CGT CCT TCT TGT ACT GGG CA | | |
| *PGK1*- forward | GCT GGA CAA GCT GGA CGT TA | | |
| *PGK1*- reverse | TGG GAC AGC AGC CTT AAT CC | | |
| *GAPDH*- forward | GCA CCA CCA ACT GCT TAG CA | | |
| *GAPDH* - reverse | TCT TCT GGG TGG CAG TGA TG | | |
| *TPI1*- forward | CAG GAA GTT CTT CGT TGG GGG | | |
| *TPI1*- reverse | GCA CAA ACC ACC TCG GTG TC | | |
| *PGAM1*- forward | TGT CAA GCA TCT GGA GGG TC | | |
| *PGAM1*- reverse | TGC ATG GGC TTG ATA GGC TT | | |
| *PGAM4*- forward | CCA AGC ATG TGG AGG GTC TC | | |
| *PGAM4*- reverse | CAC ACC GTC TCT TCA TCC CC | | |
| *PKM*- forward | CAT TAC CAG CGA CCC CAC AG | | |
| *PKM*- reverse | GAC CTG CCA GAC TTG GTG AG | | |
| *HK2*- forward | TCA CGG AGC TCA ACC ATG AC | | |
| *HK2*- reverse | GCT CCA AGC CCT TTC TCC AT | | |
| *ACTB*- forward | AGA AAA TCT GGC ACC ACA CC | | |
| *ACTB*- reverse | AGA GGC GTA CAG GGA TAG CA | | |
| *COL1A1*- forward | GTG CGA TGA CGT GAT CTG TGA | | |
| *COL1A1*- reverse | CGG TGG TTT CTT GGT CGG T | | |
| *FN1*-forward | AGG AAG CCG AGG TTT TAA CTG | | |
| *FN1*- reverse | AGG ACG CTC ATA AGT GTC ACC | | |
| *ACACA*- forward | CAT GCG GTC TAT CCG TAG GTG | | |
| *ACACA*- reverse | GTG TGA CCA TGA CAA CGA ATC T | | |
| *FASN*- forward | AAG GAC CTG TCT AGG TTT GAT GC | | |
| *FASN*- reverse | TGG CTT CAT AGG TGA CTT CCA | | |
| *SCD*- forward | TTC CTA CCT GCA AGT TCT ACA CC | | |
| *SCD*- reverse | CCG AGC TTT GTA AGA GCG GT | | |
| *DGAT2*- forward | AGC AGG TGA TCT TCG AGG AG | | |
| *DGAT2*- reverse | CAT GGG GCG AAA CCA ATG TA | | |
| *ELOVL6*- forward | AAC GAG CAA AGT TTG AAC TGA GG | | |
| *ELOVL6*- reverse | TCG AAG AGC ACC GAA TAT ACT GA | | |
| **Mouse** | | | |
| *aldoa*- forward | CTT CAA CAT GAC CCG CCT | | |
| *aldoa*- reverse | CTT TCC TAA CTC TGT CTG TTG CT | | |
| *tpi1*- forward | GGA GAG AGC CGT GCG TTT GTA | | |
| *tpi1*- reverse | AGA TGA GTT CTC CCA GGC ACT | | |
| *gapdh*- forward | TGC TGA GTA TGT CGT GGA GT | | |
| *gapdh-* reverse | GTT CAC ACC CAT CAC AAA CA | | |
| *pgk1-* forward | ACA ATG GAG CCA AGT CCG TT | | |
| *pgk1-* reverse | AAC ATC CTT GCC CAG CAG AG | | |
| *pgam1-* forward | GTT GCG AGA TGC TGG CTA TG | | |
| *pgam1- reverse* | CCG CCA TAG TGT CGC TCA TT | | |
| *actb-* forward | CAG GTC ATC ACT ATT GGC AA | | |
| *actb-*reverse | AGG TCT TTA CGG ATG TCA AC | | |
| *col1a1-* forward | GCT CCT CTT AGG GGC CAC T | | |
| *col1a1-*reverse | ATT GGG GAC CCT TAG GCC AT | | |
| *ccn2(ctgf)-* forward | GGA CAC CTA AAA TCG CCA AGC | | |
| *ccn2(ctgf)-*reverse | ACT TAG CCC TGT ATG TCT TCA CA | | |
| *fn1-* forward | GCT CAG CAA ATC GTG CAG C | | |
| *fn1-*reverse | CTA GGT AGG TCC GTT CCC ACT | | |
